# Aberrant Cerebellar–Cerebral Functional Connectivity in Children and Adolescents With Autism Spectrum Disorder

**DOI:** 10.3389/fnhum.2018.00454

**Published:** 2018-11-13

**Authors:** Ryuzo Hanaie, Ikuko Mohri, Kuriko Kagitani-Shimono, Masaya Tachibana, Junko Matsuzaki, Ikuko Hirata, Fumiyo Nagatani, Yoshiyuki Watanabe, Taiichi Katayama, Masako Taniike

**Affiliations:** ^1^Molecular Research Center for Children’s Mental Development, United Graduate School of Child Development, Osaka University, Suita, Japan; ^2^Division of Developmental Neuroscience, United Graduate School of Child Development, Osaka University, Suita, Japan; ^3^Department of Pediatrics, Osaka University Graduate School of Medicine, Suita, Japan; ^4^Department of Diagnostic and Interventional Radiology, Osaka University Graduate School of Medicine, Suita, Japan

**Keywords:** autism spectrum disorder, cerebellum, cerebral cortex, functional connectivity, resting state, MRI

## Abstract

The cerebellum, which forms widespread functional networks with many areas in the cerebral cortices and subcortical structures, is one of the brain regions most consistently reported to exhibit neuropathological features in patients with autism spectrum disorder (ASD). However, cerebellar functional connectivity (FC) studies in patients with ASD have been very sparse. Using resting state functional connectivity (rsFC) analysis, we investigated the FC of the hemispheric/vermal subregions and the dentate nucleus of the cerebellum with the cerebral regions in 36 children and adolescents [16 participants with ASD, 20 typically developing (TD) participants, age: 6–15 years]. Furthermore, an independent larger sample population (42 participants with ASD, 88 TD participants, age: 6–15 years), extracted from the Autism Brain Imaging Data Exchange (ABIDE) II, was included for replication. The ASD group showed significantly increased or decreased FC between “hubs” in the cerebellum and cerebral cortices, when compared with the TD group. Findings of aberrant FCs converged on the posterior hemisphere, right dentate nucleus, and posterior inferior vermis of the cerebellum. Furthermore, these aberrant FCs were found to be related to motor, executive, and socio-communicative functions in children and adolescents with ASD when we examined correlations between FC and behavioral measurements. Results from the original dataset were partially replicated in the independent larger sample population. Our findings suggest that aberrant cerebellar–cerebral FC is associated with motor, socio-communicative, and executive functions in children and adolescents with ASD. These observations improve the current knowledge regarding the neural substrates that underlie the symptoms of ASD.

## Introduction

Autism spectrum disorder consists of a heterogeneous group of neurodevelopmental disorders characterized by impairments in social communication and interaction, and restricted interests and repetitive behavior (American Psychiatric Association, 2013).

In addition to these core features, many studies have revealed that motor and executive dysfunctions are commonly observed in patients with ASD.

The cerebellum is one of the brain regions most consistently reported to exhibit neuropathological features in patients with ASD. These include a reduction in the number and size of Purkinje cells (Bauman and Kemper, 1985; Bailey et al., 1998; Wegiel et al., 2014b); neuroinflammation, with microglial and astroglial activation (Vargas et al., 2005); and abnormalities in the number and size of neurons in the deep cerebellar nuclei (Kemper and Bauman, 1998; Wegiel et al., 2014a). In addition, structural MRI studies on ASD have reported reduced GM volume in the posterior vermis (Webb et al., 2009; Riva et al., 2013) and Crus I and II (Riva et al., 2013; D’Mello et al., 2015). These findings have been confirmed by meta-analyses of structural MRI studies of patients with ASD (Stanfield et al., 2008; Stoodley, 2014).

Resting state functional connectivity MRI has been used to investigate FC between distributed brain regions. The rsFC MRI does not require patients to perform any tasks, and is considered to represent the status of functional brain networks (Fox and Raichle, 2007). Analysis of rsFC is performed based on spontaneous low-frequency BOLD fluctuations exhibiting temporal correlations across multiple brain regions (Biswal et al., 1995; Fox and Raichle, 2007). Previous rsFC studies in healthy adults have shown that the cerebellum forms widespread functional networks with the cerebral regions, and that different subregions of the cerebellum are functionally connected with specific regions of the cerebrum (Buckner et al., 2011; Bernard et al., 2012; Sang et al., 2012). In addition, a meta-analysis of neuroimaging studies has revealed that the cerebellum is not only involved in motor functions, but also in cognitive functions, such as executive function, and language, visuospatial, and emotional processing (Stoodley and Schmahmann, 2009).

Mounting evidence suggests that the symptoms of ASD are related to altered connectivity among diverse cortical regions (Minshew and Williams, 2007). Several rsFC studies have revealed aberrant FC in cortico-cortical (von dem Hagen et al., 2012; Lynch et al., 2013; Uddin et al., 2013) and cortico-subcortical networks (Di Martino et al., 2011; Padmanabhan et al., 2013; Nair et al., 2015) in patients with ASD. However, only a few cerebellar rsFC studies have been conducted in patients with ASD (Khan et al., 2015; Olivito et al., 2017). Khan et al. (2015) investigated cerebro-cerebellar FC using cerebral ROIs and Olivito et al. (2017) investigated FC between the dentate nucleus and the cerebral cortex. Thus, no study has comprehensively investigated which FCs between the cerebellar subregions and other brain regions are aberrant, and potential relationships to motor, socio-communicative, and executive dysfunctions in children and adolescents with ASD. Different subregions of the cerebellum have extensive FC with specific regions of the cerebral regions, and serve multiple brain functions. Thus, it is important to investigate the regional specificity of the FC between the cerebellar subregions and the cerebral regions, with particular attention toward their relationships with both motor and cognitive functions in patients with ASD. Furthermore, determining which FCs between the cerebellar subregions and other brain regions are aberrant may help provide effective therapeutic interventions, such as rTMS, for children and adolescents with ASD.

In the present study, we investigated rsFC of the hemispheric/vermal subregions and dentate nucleus of the cerebellum with the cerebral areas, at the whole brain level, in children and adolescents with ASD. In this study, we aimed: (1) to determine which FCs between the cerebellar subregions and the cerebral regions were aberrant, and (2) to examine whether such aberrant FCs were related to motor, socio-communicative, and executive functions in children and adolescents with ASD. Furthermore, we explored whether aberrant cerebellar–cerebral FCs observed in these patients would be replicated using a large sample population from the Autism Brain Imaging Data Exchange II (ABIDE II) database (Di Martino et al., 2017).

## Materials and Methods

### Participants

The *original dataset* consisted of 21 participants with ASD (21 boys, mean age: 11.3 ± 2.0 years, range: 8.1–15.1 years) and 24 TD participants (23 boys and one girl, mean age: 10.6 ± 2.4 years, range: 6.3–15.6 years). We did not use statistical methods to determine the sample size, but determined it based on previous studies (Weng et al., 2010; Di Martino et al., 2011; von dem Hagen et al., 2012; Lynch et al., 2013; Jung et al., 2014; Olivito et al., 2017). Our sample size was similar to that of previous studies.

Participants with ASD, who had no clinical history of seizures and whose ASD was not secondary to known genetic and chromosomal disorders, including trisomy 21 and tuberous sclerosis, were recruited from inpatient and outpatient pediatric programs at the Osaka University Hospital. The diagnosis of ASD was made using the criteria from the Diagnostic and Statistical Manual of Mental Disorders, Fourth Edition, Text Revision, and was further confirmed using the Autism ADOS-G (Lord et al., 2000) for all but one participant with ASD, for whom the Pervasive Developmental Disorders Autism Society Japan Rating Scale (Kamio et al., 2006) was used. One participant with ASD was treated with atomoxetine and two participants with ASD were treated with methylphenidate. These participants continued to receive their medication on the day of the scan. The TD participants were recruited from the community through advertisements and active recruitment. None of the TD participants had a history of learning, developmental, or neurological problems. The non-autistic status of the TD participants was confirmed using the Japanese version of the Autism Screening Questionnaire (Berument et al., 1999; Dairoku et al., 2004). All participants were right-handed, which was confirmed using the Edinburgh Handedness Inventory (Oldfield, 1971). Intelligence was evaluated using the Wechsler Intelligence Scale for Children, Third Edition for all but one participant with ASD, for whom the Kaufman Assessment Battery for Children was used. We chose a FSIQ threshold of ≥80 to ensure normal intelligence. Since five participants with ASD and four TD participants were excluded due to excessive maximum head motion (>3 mm) or poor MRI data quality, 16 participants with ASD (16 boys, mean age: 11.1 ± 2.0 years, range: 8.1–15.1 years), and 20 TD participants (19 boys and one girl, mean age: 10.5 ± 2.5 years, range: 6.3–15.6 years) were included in the final analysis. There were no significant differences between the two groups in age and FSIQ (Table 1). This study was approved by the Institutional Review Board of Osaka University Hospital. Written informed consent was obtained from the parents of each participant.

**Table 1:** Demographic characteristics of the participants.

	ASD	*n* = 16	TD	*n* = 20	*p-*value
			
	Mean	*SD*	Mean	*SD*	
Age (years)	11.1	2.0	10.5	2.5	0.401
FSIQ^a^	107.8	12.1	110.0	10.4	0.561
VIQ^a^	111.2	14.6	114.2	12.6	0.510
PIQ^a^	103.7	13.5	103.0	11.3	0.859
ADOS-G subscales^b^					
Communication	3.6	1.1			
Reciprocal social interaction	7.3	2.2			
Stereotyped behaviors and	0.7	0.9			
restricted interests					
Total	10.8	2.7			
PARS^c^	19.0				
ASQ-J			4.0	4.1	
Head motion (mean FD)	0.15	0.06	0.11	0.04	0.140
Valid scan images	131.9	10.6	136.5	5.4	0.962

*ASD, autism spectrum disorder; TD, typically developing controls; FSIQ, full-scale intelligence quotient; VIQ, verbal intelligence quotient; PIQ, performance intelligence quotient; ADOS-G, Autism Diagnostic Observation Schedule – Generic; PARS, Pervasive Developmental Disorders Autism Society of Japan Rating Scale (cutoff ≥9); ASQ-J, Japanese version of the Autism Screening Questionnaire (cutoff ≥13); FD, framewise displacement; SD, standard deviation.*

*^*a*^Scores for the mental processing composite, sequential processing scale, and simultaneous processing scale of the Kaufman Assessment Battery for Children were entered as the FSIQ, VIQ, and PIQ, respectively, for one participant with ASD.*

*^*b*^Data from ADOS-G were available for 15 participants with ASD.*

*^*c*^Data from PARS were available for one participant with ASD.*

### Behavioral Assessments

The M-ABC 2 was used to assess motor function of the participants (Henderson et al., 2007). The M-ABC 2 includes eight subtests, which assess three components of motor function: manual dexterity, ball skills, and static and dynamic balance. In addition, a total test score is obtained. These scores are converted to standard scores, which range from 1 to 19, with higher scores indicative of better motor function. The M-ABC 2 has three modules for the following age ranges: 3–6, 7–10, and 11–16 years. The appropriate module was used for each participant.

To assess socio-communicative function, we used the SRS 2, which is a validated parent- or teacher-completed questionnaire used to assess social impairment and ASD severity (Constantino and Gruber, 2012). We administered a parent-completed questionnaire. The SRS 2 consists of a set of 65 items, and produces scores for five domains: social awareness, social cognition, social communication, social motivation, and restricted interests and repetitive behavior. Scores for social awareness, social cognition, social communication, and social motivation are combined to generate the SCI. We also calculated the total score. Higher scores on the SRS 2 indicate greater severity of socio-communicative impairment.

In addition, we also used the BRIEF to assess the executive function of the participants (Gioia et al., 2000). The BRIEF is a parent- or teacher-completed questionnaire assessing behavior associated with executive function. We administered a parent-completed questionnaire. The BRIEF consists of a set of 86 items, and generates scores for eight subdomains of executive function, an overall score GEC, and two index scores (BRI and MI). The BRI is composed of three subdomain scores (inhibit, shift, and emotional control), and the MI is composed of five subdomain scores (initiate, working memory, plan/organize, organization of materials, and monitor). The BRI and MI are combined to obtain the GEC. Higher scores on the BRIEF indicate greater severity of executive dysfunction. Behavioral assessment data were available for a subset of participants (M-ABC 2: ASD = 8, TD = 15; SRS: ASD = 10, TD = 17; BRIEF: ASD = 13, TD = 17).

### Replication Dataset

We obtained the *replication dataset* from ABIDE II.^1^ The ABIDE II consists of datasets from 487 individuals with ASD and 557 controls collected across 16 imaging sites. The original studies included in ABIDE II received approval from each site’s Institutional Review Board.

We selected imaging sites that reported scores on the SRS (Constantino and Gruber, 2005), SRS 2 (Constantino and Gruber, 2012), and the BRIEF (Gioia et al., 2000). The imaging sites that met these criteria were the GU, KKI, and NYU Langone Medical Center: Sample 1 (NYU_1), NYU: Sample 2 (NYU_2), and SDSU (GU dataset: ASD = 51, TD = 55; KKI dataset: ASD = 56, TD = 155; NYU_1 dataset: ASD = 48, TD = 30; NYU_2 dataset: ASD = 27, TD = 0; SDSU dataset: ASD = 33, TD = 25). From these five imaging sites, in order to collect samples similar to our original samples, we selected subjects who met following criteria: boys with FSIQ ≥ 80, age range: 6–16 years, right handed, with scores recorded on the SRS, SRS 2, and BRIEF (GU dataset: ASD = 37, TD = 23; KKI dataset: ASD = 29, TD = 79; NYU_1 dataset: ASD = 18, TD = 24; NYU_2 dataset: ASD = 9, TD = 0; SDSU dataset: ASD = 13, TD = 11). Diagnosis of ASD was made using ADOS-G or ADOS-2 (Lord et al., 2012). After checking the images, we selected only the participants with complete cerebellar coverage (GU dataset: ASD = 6, TD = 5; KKI dataset: ASD = 28, TD = 71; NYU_1 dataset: ASD = 11, TD = 18; NYU_2 dataset: ASD = 0, TD = 0; SDSU dataset: ASD = 13, TD = 11). Since no participants in the NYU_2 dataset had complete cerebellar coverage, this dataset was excluded. In addition, participants with excessive maximum head motion (>3 mm) or failure of normalization were excluded.

The final replication dataset consisted of 42 boys with ASD (mean age: 10.7 ± 1.8 years, range: 7.2–14.4 years) and 88 TD boys (mean age: 10.3 ± 1.7 years, range: 6.4–15.3 years; GI dataset: ASD = 4, TD = 4; KKI dataset: ASD = 17, TD = 56; NYU_1 dataset: ASD = 11, TD = 18; SDSU dataset: ASD = 10, TD = 10). Within each site, there were no significant differences between the two groups in age and FSIQ (except for one site). Across sites, there was a significant difference between the two groups in FSIQ, but not in age. Supplementary Table S1 presents the demographic data for each imaging site and the entire sample population of the replication dataset.

For the replication dataset, the SRS, SRS 2, and BRIEF scores were available, but M-ABC-2 scores were not available, nor were SCI scores on the SRS 2.

### MRI Data Acquisition

For the original dataset, all images were acquired using a 3-T GE MR system (Signa Excite HDxt; GE Healthcare, Milwaukee, WI, United States). One-hundred and fifty whole-brain functional volumes were obtained, which included the entire cerebellum. We used a gradient echo, echo planar imaging pulse sequence with 40 axial slices (repetition time = 2,000 ms, echo time = 30 ms, flip angle = 90°, field of view = 220 mm × 220 mm, matrix size = 64 × 64, slice thickness = 3.5 mm, voxel size = 3.4 mm × 3.4 mm × 3.5 mm). The echo planar imaging data were acquired for 5 min. During the scan, participants were instructed to rest with their eyes closed but to stay awake. Three-dimensional T1-weighted structural images were obtained using spoiled gradient-recalled acquisition in steady state sequence (repetition time = 10.1 ms, echo time = 3.0 ms, flip angle = 18°, field of view = 220 m × 220 mm, matrix size = 320 × 256, slice thickness = 1.4 mm, voxel size = 0.68 mm × 0.85 mm × 1.4 mm, number of slices = 128, number of excitations = 1). Foam pillows and cushions were used to minimize participants’ head movement during the scans.

For the replication dataset, complete details of the MRI scanning parameters for each imaging site are provided in the original publication (Di Martino et al., 2017).

### fMRI Data Analysis

All analyses were performed using the CONN toolbox^2^ and the Statistical Parametric Mapping 12 software.^3^ Preprocessing for functional images consisted of realignment for motion correction, slice-timing correction, normalization to the standard MNI space, and smoothing with a 4-mm full width at half-maximum filter.

To remove the effects of head motion artifacts and physiological noise, which can induce spurious correlations and influence the outcome of rsFC analysis (Power et al., 2012, 2014; Yan et al., 2013), Artifact Detection Tools,^4^ and the CompCor method (Whitfield-Gabrieli and Nieto-Castanon, 2012) were used in the CONN toolbox. Using Artifact Detection Tools, images with FD larger than 0.5 mm or signal intensity changes greater than three standard deviations were defined as outlier images. The CompCor method was used to estimate and remove motion and physiological noise without regressing out the global signal. The CompCor method has better sensitivity and specificity to detect FC across multiple brain regions than the global signal regression method (Whitfield-Gabrieli and Nieto-Castanon, 2012). Structural images were normalized and segmented into GM, white matter, and cerebrospinal fluid regions. Using principal components analysis, five principal components were extracted from subject-specific segmented white matter and cerebrospinal fluid, which were used as temporal confounding factors and removed from the BOLD functional data in the time-series linear regression. In addition, the six motion parameters from the realignment, and their temporal derivatives along with outlier images, were used as nuisance covariates in the time-series linear regression. Data sets were band-pass filtered from 0.008 to 0.09 Hz.

**FIGURE 1:**
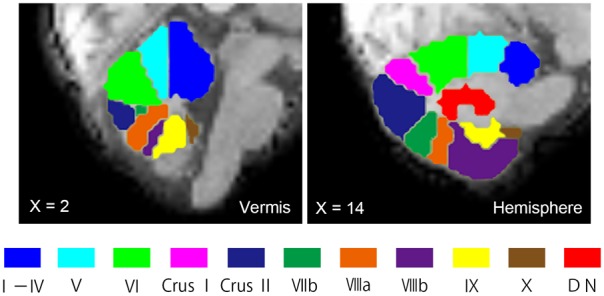
Seed ROIs in the cerebellum used for FC analysis. Seed ROIs for the vermis, the hemisphere, and the dentate nucleus of the cerebellum were created. The ROIs are overlaid onto sagittal images of the representative participant’s normalized T1-weighted images. An ROI for the vermis Crus I is not shown in this figure, as it does not appear in this vermal slice (*X* = 2). ROIs, regions of interest; FC, functional connectivity; DN, dentate nucleus.

Seed ROIs in the cerebellum were created using the probabilistic MR Atlas of the human cerebellum (Diedrichsen, 2006; Diedrichsen et al., 2009, 2011) and MRIcron.^5^ Among the several types of atlases in the probabilistic MR Atlas, the “MNIsegment version” was used in this study. With reference to a previous rsFC study of the cerebellum (Sang et al., 2012), we created seed ROIs for 20 lobules (10 per hemisphere: lobules I–IV, V, VI, Crus I, Crus II, VIIb, VIIIa, VIIIb, IX, and X), eight vermis regions (vermis VI, Crus I, Crus II, VIIb, VIIIa, VIIIb, IX, and X), and the bilateral dentate nuclei (Figure 1). The ROIs for lobules I–IV and V included the vermis. Since little is known about cerebellar connectivity in children and adolescents, and we aimed to determine which FCs between the cerebellar subregions and other brain regions are aberrant, we used all 30 ROIs as seed ROIs in this study. We confirmed the correspondence between the cerebellar seed ROIs and individual structural images of the cerebellum using MRIcron.

In the first-level analysis, the average BOLD time-series was computed across all voxels within each cerebellar seed ROI, and Pearson’s correlation coefficients were calculated between that time-series and the time-series of all other voxels in the brain. Correlation coefficients were converted to normally distributed *Z*-scores using Fisher’s transformation, resulting in a connectivity map for each individual, which was entered into the second-level analysis. The average BOLD time-series within each cerebellar seed ROI was extracted from unsmoothed data to decrease potential spillage of the BOLD signal from nearby regions. In addition, seed ROIs’ voxels, from which the average BOLD signal was extracted, were restricted to the GM using the segmented GM mask. In the second-level analysis, group differences in connectivity maps were examined using two sample *t*-tests. There was no significant group difference in mean FD scores (see below). Nonetheless, because previous studies have shown that comparisons between groups with subtly different levels of head motion yielded group difference in rsFCs (Van Dijk et al., 2012; Power et al., 2014), we included mean FD scores as a nuisance covariate in the second-level analyses. In addition, FSIQ was also included as a nuisance covariate. All results were reported using a voxel-height threshold set at uncorrected *p* < 0.001, with a cluster-extent threshold at a FDR-corrected *p* < 0.05.

In the replication dataset, we performed the same preprocessing procedures as described above, and tested whether our findings of the 12 aberrant cerebellar–cerebral FCs in the original dataset could be replicated using ROI-to-ROI analysis. We created cerebral ROIs from clusters with significant group differences, and computed the ROI-to-ROI correlation coefficients between these cerebral ROIs and a set of cerebellar ROIs with significant group differences in the original dataset. Group differences in the ROI-to-ROI FCs were examined using two sample *t*-tests. For the same reason described above, mean FD scores were included as a nuisance covariate in *t*-tests. In addition, FSIQ and imaging sites were included as nuisance covariates. Bonferroni correction was performed for multiple comparisons (*p* = 0.004).

### Correlation Analyses

In the ASD group, to investigate relationships between the strengths of the FCs and behavioral measurements, correlation analyses were performed between FC values showing significant group differences and scores from the M-ABC 2, SRS 2, and BRIEF tests. Correlation analyses were performed for the subset of participants with ASD whose data were available (M-ABC 2 = 8, SRS 2 = 10, BRIEF = 13). Pearson’s correlation coefficients were used in all correlation analyses.

Although there were several test scores in each test, we used representative scores of each test to reduce the number of statistical tests in correlation analyses. In the M-ABC 2, total test scores were used for the correlation analyses. In the SRS 2 and BRIEF, the total and GEC scores were used, respectively. In addition, to investigate relationships between FC strength and the severity of ASD symptoms, correlation analyses were performed between the FC values showing significant group differences and total scores on the ADOS-G. Since different ADOS-G modules were used across participants (number of participants: module 1 = 1, module 2 = 2, and module 3 = 12), correlation analyses were performed only for participants who were administered module 3.

For the replication dataset, the same correlation analyses were performed in the ASD group except for M-ABC 2. Regarding the total score of ADOS, correlation analyses for ADOS-G and ADOS-2 were conducted separately. These correlation analyses were performed only for participants who were administered module 3 (ADOS-G = 16, ADOS-2 = 31; note that eight participants were administered both ADOS-G and ADOS-2).

## Results

### Group Differences in Behavioral Measurements

Independent samples *t*-tests were used to compare behavioral measurements between ASD and TD groups. In the original dataset, the ASD group had poorer performance on the motor tests than the TD group, except for the static and dynamic balance tests of the M-ABC 2 (total test score, *p* = 0.001). The ASD group had lower scores for manual dexterity (*p* = 0.017) and ball skills (*p* = 0.0007, Table 2). The ASD group had poorer socio-communicative function than the TD group, as indicated by higher scores on all SRS 2 subscales (*p* < 0.02), the SCI (*p* = 0.00003), and total score (*p* = 0.00001, Table 2). On the BRIEF, the ASD group showed poorer executive function than the TD group, as indicated by higher scores in several subdomains (inhibit, shift, initiate, working memory, and monitor: *p* < 0.02), the BRI (*p* = 0.008), the MI (*p* = 0.024), and the GEC (*p* = 0.009, Table 2).

**Table 2:** Behavioral measurement scores.

	ASD			TD			*p-*value
	Mean	*SD*	Range	Mean	*SD*	Range	
**Movement Assessment Battery for Children 2^a^**				
Manual dexterity	9.6	3.8	3–14	13.3	2.8	10–19	0.017*
Ball skills	8.0	2.6	5–12	12.3	2.5	8–16	0.001**
Static and dynamic balance	10.8	3.0	6–14	12.4	1.9	9–14	0.119
Total test score	9.4	3.4	5–14	14.0	2.5	10–19	0.001**
**Social Responsiveness Scale 2^b^**				
Social awareness	59.8	11.1	38–76	48.2	9.1	35–67	0.00706**
Social cognition	57.4	8.2	39–68	47.5	5.9	39–61	0.00124**
Social communication	58.0	8.0	45–68	45.5	4.8	38–57	0.00003***
Social motivation	54.9	9.2	40–73	47.3	5.2	38–60	0.01069
Restricted interests and repetitive behavior	54.6	6.3	46–66	45.6	4.3	41–55	0.00017***
Social communication and interaction index	58.9	6.9	46–66	46.2	5.7	36–61	0.00003***
Total score	58.2	6.4	46–66	46.1	4.9	37–58	0.00001***
**Behavior Rating Inventory of Executive**				
**Function^c^**							
Inhibit	50.0	10.8	36–72	41.8	4.4	37–58	0.008**
Shift	55.0	10.9	41–74	43.4	8.6	36–68	0.003**
Emotional control	49.1	11.0	37–70	44.9	10.4	36–73	0.295
Initiate	53.8	10.7	36–73	44.2	8.5	35–65	0.010*
Working memory	53.3	8.5	38–69	43.1	7.4	30–60	0.001**
Plan/organize	51.0	12.1	37–75	46.9	9.2	33–70	0.298
Organization of materials	49.7	9.8	34–67	47.1	9.6	33–69	0.478
Monitor	54.8	11.4	38–73	42.8	9.0	28–63	0.003**
Behavioral regulation index	51.0	11.7	37–72	41.1	7.4	35–66	0.008**
Metacognition index	52.9	11.1	37–75	44.3	8.5	30–67	0.024*
Global executive composite	52.5	11.7	36–76	42.4	8.3	30–68	0.009**

*^*a*^Data from the Movement Assessment Battery for Children 2 were available for eight participants with ASD and 15 TD participants.*

*^*b*^Data from the Social Responsiveness Scale 2 were available for 10 participants with ASD and 17 TD participants.*

*^*c*^Data from the Behavior Rating Inventory of Executive Function were available for 13 participants with ASD and 17 TD participants.*

*ASD, autism spectrum disorder; TD, typically developing.*

*^∗^*p* < 0.05, ^∗∗^*p* < 0.01, ^∗∗∗^*p* < 0.0005.*

For the replication dataset, the same analyses were performed, except for the M-ABC-2. Results of group comparisons were very similar to the original dataset. The ASD group had poorer socio-communicative function than the TD group, as indicated by higher scores on all subscales (*p* < 0.00001) and total scores (*p* < 0.00001) of the SRS and SRS 2. The ASD group also had poorer executive function than the TD group, as indicated by higher scores in all subdomains (*p* < 0.00001), the BRI (*p* < 0.00001), MI (*p* < 0.00001), and GEC (*p* < 0.00001), of the BRIEF. Supplementary Table S2 shows the results of group comparisons of behavioral measurements for the replication dataset.

### Group Differences in Head Motion

Mann–Whitney *U* tests were used to compare mean FD scores and the number of valid scan images between ASD and TD groups. There were no significant differences between groups in mean FD scores (*p* = 0.140) and the number of valid scan images (*p* = 0.962) in the original dataset (Table 1). The minimum number of valid scan images was 111 across groups.

For the replication dataset, there were no significant differences between groups in mean FD scores (*p* = 0.152) and the number of valid scan images (*p* = 0.950). The minimum number of valid scan images was 90 across groups. Supplementary Table S1 presents the data of head motion for each imaging site and the entire sample population of the replication dataset.

### Patterns of Cerebellar Functional Connectivity

We found both positive and negative FCs between the cerebellum and cerebral regions in our original dataset. Regarding positive FCs, patterns of cerebellar FCs in both ASD and TD groups were largely similar to those of previous FC studies in healthy adults (Buckner et al., 2011; Bernard et al., 2012, 2013, 2014). In the hemispheric lobules, we observed positive FCs between the anterior cerebellum and the cerebral sensorimotor cortices, as well as between the posterior cerebellum and cognitive regions, including the prefrontal, parietal, and temporal association cortices. We also identified FCs between several hemispheric lobules and subcortical structures, such as the thalamus, basal ganglia, amygdala, hippocampus, and brainstem. Several FCs were found between the posterior vermis and sensorimotor or cognitive regions in the cerebral cortices. In addition, the posterior vermis was connected to the subcortical structures, such as the thalamus, basal ganglia, amygdala, hippocampus, and brainstem. In the dentate nucleus, we observed FCs with both motor and cognitive regions. Overall, in the TD group, the cerebral regions positively connected with the cerebellar subregions were more widespread than those of the ASD group. The supplementary figures show patterns of cerebellar positive FC in the ASD and TD groups (Supplementary Figures S1–S6).

Regarding negative FCs, although we observed several negative FCs between the cerebellum and cerebral regions in both the groups, because previous FC studies in healthy adults did not report negative cerebellar FCs, we could not compare our results with those of previous studies. However, we found several significant features of negative cerebellar FCs. Namely, compared to the positive cerebellar FCs, the cerebral areas negatively connected with the cerebellar subregions were narrower, and there were no negative FCs for several cerebellar subregions, such as the right lobule VIIIa and VIIIb in both the groups. In addition, it was difficult to identify consistent patterns in the negative cerebellar FCs compared with the positive cerebellar FCs across groups. Nonetheless, there were negative FCs between the lobules I–IV and the SPL, and between the lobule V and the IPL in both ASD and TD groups. In addition, for the lobule VI and Crus I, we observed negative FCs with the precuneus and AG in both groups, which are included in the default mode network. The supplementary figures show patterns of cerebellar positive FC in the ASD and TD groups (Supplementary Figures S7–S12).

### Group Differences in Cerebellar Functional Connectivity

**FIGURE 2:**
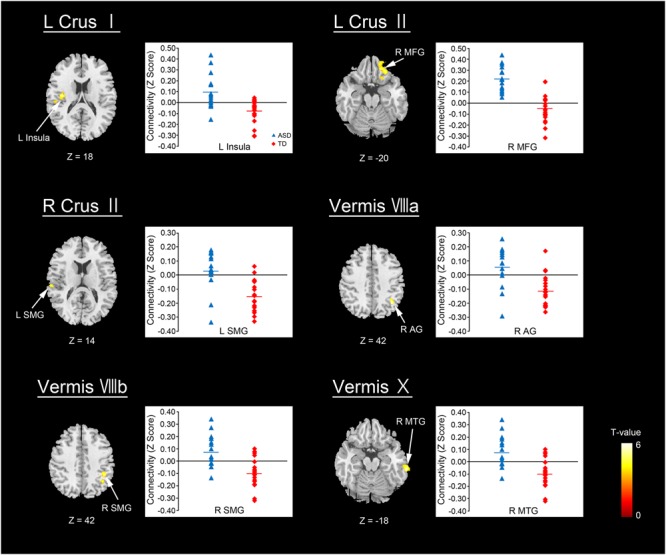
Brain regions with increased FC in the ASD group. Here, we show the cerebral areas showing increased FC with the cerebellar hemisphere and vermis in the ASD group relative to the TD group. All results are thresholded using a voxel-height threshold at uncorrected *p* < 0.001 with cluster-extent threshold at false discovery rate-corrected (*p* < 0.05). Images were created using the xjview toolbox (http://www.alivelearn.net/xjview). ASD, autism spectrum disorder; TD, typically developing; MFG, middle frontal gyrus; SMG, supramarginal gyrus; AG, angular gyrus; MTG, middle temporal gyrus; L, left; R, right.

The ASD group showed significantly increased or decreased FCs between the cerebellar subregions and cerebral cortices, relative to the TD group in the original dataset. The increased FCs were between the following regions: the left Crus I and left insula, left Crus II and right MFG, right Crus II and left SMG, vermis VIIIa and right AG, vermis VIIIb and right SMG, and vermis X and right MTG (Figure 2 and Table 3). In general, in the brain regions showing increased FCs in the ASD group, there was significant positive FC between these brain regions, whereas there was significant negative FC between the same regions in the TD group, except for the FC between the right Crus II and left SMG. In the right Crus II, significant negative FC with the left SMG was found in the TD group, but the FC value between these two regions was not significantly different from zero in the ASD group.

**Table 3:** Group differences in functional connectivity in the cerebellar seed regions of interest (ROIs).

Seed ROIs	Region	BA	Cluster size	MNI coordinate			*T*-value
				*X*	*Y*	*Z*
	**ASD > TD**						
Left Crus I	**Left insula**	13	144	–40	–12	18	5.44
	Left supramarginal gyrus			–52	–24	20	4.11
Left Crus II	**Right middle frontal gyrus**		229	16	40	–20	4.85
	Right inferior frontal gyrus	11		24	36	–20	4.79
	Right inferior frontal gyrus			14	22	–18	4.61
Right Crus II	**Left supramarginal gyrus**	40	89	–62	–24	14	4.78
	Left supramarginal gyrus			–54	–22	20	4.03
Vermis VIIIa	**Right angular gyrus**	40	117	38	–58	42	5.98
	Right angular gyrus			32	–52	38	5.25
Vermis VIIIb	**Right supramarginal gyrus**	40	183	42	–42	42	5.16
	Right inferior parietal lobule			38	–54	42	4.54
Vermis X	**Right middle temporal gyrus**		112	66	–32	–18	5.53
							
	**ASD < TD**						
Right lobule IX	**Right cerebellar lobule VI**		78	32	–56	–34	4.91
Vermis VI	**Left middle frontal gyrus**		381	–22	18	52	6.31
	Left middle frontal gyrus	8		–32	28	46	5.34
	Left middle frontal gyrus	8		–24	24	46	4.45
	**Left inferior temporal gyrus**		96	–52	–34	–20	4.26
	Left middle temporal gyrus			–56	–38	–14	3.97
	**Right medial frontal gyrus**	8	72	6	26	46	4.70
	Right medial frontal gyrus	9		4	34	38	4.60
Right dentate nucleus	**Left inferior frontal gyrus**		105	–20	34	–24	5.61
	Left inferior frontal gyrus	47		–18	16	–24	3.75
	**Left inferior parietal lobule**	40	85	–48	–48	54	4.29
	Left inferior parietal lobule	40		–54	–42	52	4.07
	Left inferior parietal lobule	40		–56	–50	46	4.03

*ASD, autism spectrum disorder; TD, typically developing controls; MNI, Montreal Neurological Institute; BA, Brodmann area.*

*Regions in bold font indicate the peak voxel in a particular cluster. All results are reported using a voxel-height threshold at uncorrected *p* < 0.001 with cluster-extent threshold at false discovery rate-corrected *p* < 0.05.*

**FIGURE 3:**
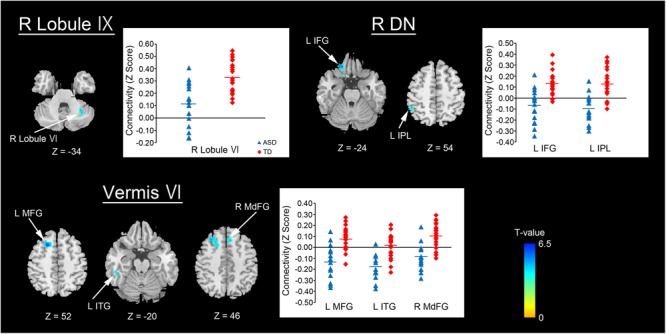
Brain regions with decreased FC in the ASD group. Here, we show the cerebral and cerebellar areas showing decreased FC with the cerebellar hemisphere, the vermis, and the dentate nucleus of the cerebellum in the ASD group relative to the TD group. All results are thresholded using a voxel-height threshold at uncorrected *p* < 0.001 with cluster-extent threshold at false discovery rate-corrected *p* < 0.05. Images were created using the xjview toolbox (http: //www.alivelearn.net/xjview). FC, functional connectivity; ASD, autism spectrum disorder; DN, dentate nucleus; IFG, inferior frontal gyrus; IPL, inferior parietal lobule; MFG, middle frontal gyrus; ITG, inferior temporal gyrus; MdFG, medial frontal gyrus; L, left; R, right.

By contrast, decreased FCs were identified between the following regions: the right lobule IX and right lobule VI; vermis VI and left MFG, left ITG, and right MdFG; right dentate nucleus and left IFG, left IPL (Figure 3 and Table 3). In general, in the brain regions showing decreased FCs in the ASD group, there was significant negative FC between these brain regions, whereas there was significant positive FC in the same regions in the TD group, except for FCs between the right lobule IX and right lobule VI, and between the vermis VI and left ITG. Both ASD and TD groups had significant positive FC between the right lobule IX and right lobule VI. In the vermis VI, significant negative FC with the left ITG was found in the ASD group, but this was not significant in the TD group.

**FIGURE 4:**
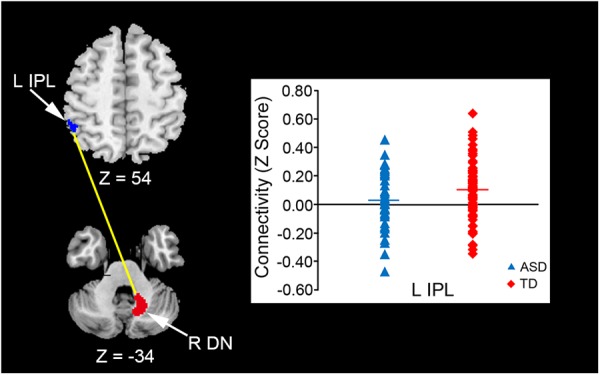
Decreased FC between the right dentate nucleus and left IPL in the ASD group. An ROI-to-ROI analysis revealed that the ASD group had significantly decreased FC between the right dentate nucleus and left IPL relative to the TD group in the replication dataset (*p* = 0.039). FC, functional connectivity; ASD, autism spectrum disorder; ROI, region of interest; TD, typically developing; IPL, inferior parietal lobule; DN, dentate nucleus; L, left; R, right.

For the replication dataset, compared with the TD group, the ASD group showed significantly decreased FC between the right dentate nucleus and left IPL (*p* = 0.039, Figure 4). Significant positive FC between these brain regions was found in the TD group, but not in the ASD group. Additionally, the ASD group showed a trend toward significantly decreased FC between the vermis VI and left MFG (*p* = 0.071). After Bonferroni correction for multiple comparisons, these differences did not remain significant.

### Correlations With Behavioral Measurements

In the ASD group, we investigated the correlations between motor, socio-communicative, and executive functions, and the severity of ASD symptoms and cerebellar FC value.

**FIGURE 5:**
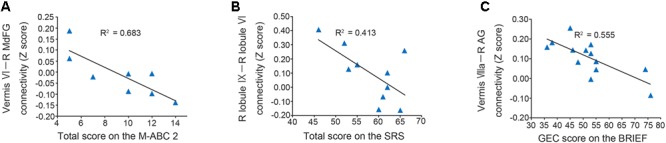
Scatter plots of correlation analyses of FC values and behavioral measurements in the ASD group. **(A)** FC strength between the vermis VI and right MdFG was negatively correlated with total test score on the M-ABC 2 (*r* = –0.826, *p* = 0.011). **(B)** FC strength between the right lobule IX and right lobule VI was negatively correlated with total score on the SRS 2 (*r* = –0.643, *p* = 0.045). **(C)** FC strength between the vermis VIIIa and right AG was negatively correlated with GEC score on the BRIEF (*r* = –0.745, *p* = 0.003). FC, functional connectivity; ASD, autism spectrum disorder; MdFG, medial frontal gyrus; M-ABC 2, Movement Assessment Battery for Children 2; SRS 2, Social Responsiveness Scale 2; AG, angular gyrus; GEC, global executive composite; BRIEF, Behavior Rating Inventory of Executive Function.

In the original dataset, the FC value between the vermis VI and right MdFG was negatively correlated with total test score on the M-ABC 2 (*r* = -0.826, *p* = 0.011; Figure 5A). This result indicates that the decreased FC between these regions was associated with better motor performance. In the correlation analyses of FC value and socio-communicative function, the FC value between the right lobule IX and right lobule VI had a negative correlation with the total score on the SRS 2 (*r* = -0.643, *p* = 0.045; Figure 5B), indicating that increased connectivity between these regions was associated with better socio-communicative function. In the correlation analyses of FC value and executive function, the GEC score of the BRIEF had a negative correlation with the FC value between the vermis VIIIa and right AG (*r* = -0.745, *p* = 0.003; Figure 5C). This result indicates that the increased FC between these regions was associated with better executive function. There was no correlation between cerebellar FC value and total score on the ADOS-G. Since there were no correlations among cerebellar FC value, age, FSIQ, or mean FD score, we did not include these variables as nuisance covariates.

For the replication dataset, FC values between the right dentate nucleus and left IPL, which were the only FC values that differed significantly between ASD and TD groups, were not correlated with the behavioral measurements or ADOS scores.

## Discussion

This is the first study to reveal aberrant FCs between the cerebellum and cerebral regions using cerebellar seed ROIs in children and adolescents with ASD. Compared with the TD group, the ASD group had both increased and decreased FCs of the cerebellum regions with cerebral areas, and these aberrant FCs were correlated with several behavioral measures. Furthermore, our findings were partially replicated in the larger dataset. Results of this study concur well with those of previous diffusion tensor imaging studies reporting abnormal altered microstructural properties in the SCP and MCPs, as indicated by lower fractional anisotropy in patients with ASD (Catani et al., 2008; Shukla et al., 2010; Hanaie et al., 2013). These diffusion tensor imaging studies suggest the presence of abnormal structural connectivity between the cerebellum and cerebral regions.

We found increased FCs between the hemispheric subregions of the cerebellum and cerebral cortices in the ASD group, except for one decreased FC. Findings of aberrant FCs converged primarily on the posterior hemisphere of the cerebellum, namely, the Crus I, Crus II, and lobule IX. These results are consistent with those of previous volumetric studies reporting GM volume reduction in the Crus I/II (Riva et al., 2013; D’Mello et al., 2015) and correlations between GM volume in the lobule IX and social symptom severity in patients with ASD (D’Mello et al., 2015). A recent neuropathological study in patients with ASD reported reduced density of Purkinje cells in the Crus I/II (Skefos et al., 2014). In addition, a neurochemical study has found reduced mRNA levels of glutamic acid decarboxylase 67, which is an enzyme involved in the synthesis of γ-aminobutyric acid (GABA) (Fatemi et al., 2002), in Purkinje cells of the Crus II in patients with ASD (Yip et al., 2007).

The cerebellar posterior hemisphere, which showed aberrant FCs with cerebral cortices, is involved in a variety of cognitive functions. The Crus I/II are involved in language processing and executive function (Habas et al., 2009; Stoodley and Schmahmann, 2009). Although the functional role of the lobule IX remains unclear, it might be involved in social cognition by cooperation with the default mode network in the cerebral regions (Habas et al., 2009). We found decreased FC between the lobule IX and lobule VI, and this was correlated with poorer socio-communicative function in the ASD group. Since the lobule VI is involved in language processing (Stoodley and Schmahmann, 2009), the results of our correlation analysis are consistent with the functional role of the lobule IX.

We observed aberrant FCs between areas of the posterior vermis, such as the vermis VI, VIIIa, VIIIb, and X, and the cerebral cortices. These findings are consistent with those of previous volumetric studies reporting GM volume reduction in the posterior vermis in patients with ASD (Stanfield et al., 2008; Webb et al., 2009; Riva et al., 2013). In addition, we found negative correlations between: (1) GEC scores on the BRIEF and FC values between the vermis VIIIa and right AG, and (2) total test scores on the M-ABC 2 and FC values between the vermis VI and right MdFG. The former result indicates that increased FC between these regions was associated with better executive function, and the latter indicates that decreased FC between these regions was associated with better motor function. The FC between the vermis VIIIa and right AG increased, and that between the vermis VI and right MdFG decreased in the ASD group relative to the TD group. Additionally, in TD individuals, the posterior vermis is known to be involved in oculomotor control and affective processing by cooperation with the brainstem and limbic system, respectively (Grimaldi and Manto, 2012; D’Mello and Stoodley, 2015). Therefore, the results of these correlation analyses are counterintuitive. Findings of increased FC between the vermis VIIIa and right AG, and decreased FC between the vermis VI and right MdFG might imply the existence of compensatory mechanisms in cerebellar–cerebral circuitry in patients with ASD.

In the dentate nuclei, decreased FC with the cerebral regions converged on the right dentate nucleus. Furthermore, decreased FC between the right dentate nucleus and left IPL was observed in the replication dataset. Such convergence on the right dentate nucleus is also consistent with the results of our previous diffusion tensor imaging study as well as another study that reported reduced fractional anisotropy in the right SCP, whose fibers originate from the right dentate nucleus, in patients with ASD (Catani et al., 2008; Hanaie et al., 2013). In addition, an MRI tractography study has found that both the numbers of streamlines and fractional anisotropy were reduced in the pathway connecting the right posterior lateral cerebellar cortex with the right dentate nucleus in patients with ASD (Jeong et al., 2014). Furthermore, a number of neuropathological findings in the dentate nuclei of patients with ASD have been documented, including decreased neuron size (Wegiel et al., 2014a), discontinuation of the dentate ribbon (Bailey et al., 1998), and reduced glutamic acid decarboxylase 65 mRNA levels in the subpopulations of the dentate nuclei in patients with ASD (Yip et al., 2009). Thus, neuropathological abnormalities in the dentate nuclei may be associated with our findings of decreased FCs.

The cerebral regions showing aberrant FCs with the cerebellum and the posterior hemisphere, vermis, and dentate nucleus of the cerebellum are considered to be “hubs,” which form part of a complex network that supports cognition and behavior in the human brain (Achard et al., 2006;Cole M.W. et al., 2010; Hwang et al., 2013; Collin et al., 2014). For example, the insula and AG serve as hubs in the salience network and default mode network, respectively (Uddin, 2015; Padmanabhan et al., 2017). The MFG and SMG also form parts of the executive control network (Seeley et al., 2007; Habas et al., 2009). “Hubs” play an important role in facilitating communication among distributed brain networks, maintaining efficient information flow and integration (Hwang et al., 2013). Therefore, aberrant FCs between these cerebral regions and the cerebellum might lead to disruption of cooperation among brain networks in patients with ASD; however, such an idea is speculative and requires further verification.

Since results of rsFC analysis do not represent the directionality of information flow between two brain regions (Cole D.M. et al., 2010), it is difficult to infer the source of aberrant FCs. However, aberrant FCs between the cerebellum and cerebral cortices may be attributed to several pathological changes within the cerebellum. In addition to the decreased number of inhibitory Purkinje cells and other neuropathological abnormalities of the dentate nuclei mentioned above, biochemical studies of postmortem brain samples from patients with ASD have reported alterations in the cerebellar GABAergic inhibitory system, and suggest that there exists an imbalance between excitatory and inhibitory circuits in the cerebellum, which may disrupt inputs to the dentate nuclei (Blatt, 2005; Blatt and Fatemi, 2011; Hanaie et al., 2013). This could subsequently affect the outputs of the dentate nuclei, and in turn, lead to abnormal activity in the cerebral cortices as well as aberrant FCs between the cerebellum and cerebral cortices in patients with ASD.

Our findings of the convergence of aberrant FCs primarily on the posterior hemisphere, the right dentate nucleus, and the posterior vermis of the cerebellum can help provide effective therapeutic interventions for children with ASD. Recently, rTMS has been used as a therapeutic intervention in patients with ASD, and various brain regions such as the dorsal lateral prefrontal, dorsal medial prefrontal, and primary motor cortices have been targeted (Oberman et al., 2015). To date, there have been no TMS interventions targeting the cerebellum in patients with ASD. Recent TMS studies have shown that changes in cerebellar activity may influence both inhibitory and excitatory activity in cerebral regions (Daskalakis et al., 2004; Koch, 2010). Therefore, rTMS targeting the posterior hemisphere, the right dentate nucleus, and the posterior vermis of the cerebellum may provide a novel therapeutic approach to changing the activities of other brain regions and improving behavioral problems in children and adolescents with ASD.

Our study has several limitations. First, since data from behavioral assessments were available for only a subset of the participants in the original dataset, interpretation of the correlation analyses between behavioral assessments and FC values needs caution. Second, our findings were not fully replicated in the replication dataset. This could be due to heterogeneity of the participants with ASD in the replication dataset. Our inclusion criteria for the replication dataset included complete cerebellar coverage and availability of SRS and BRIEF scores. In the ABIDE II, few subjects met these inclusion criteria. Therefore, it is possible that our replication dataset was not sufficiently large to overcome the heterogeneity in ASD. Regarding comorbidity, a large number of participants with ASD exhibiting comorbidities, such as attention deficit hyperactivity disorder, were included in our replication dataset (see Supplementary Table S1). To the best of our knowledge, although no prior studies have compared cerebellar FCs in patients with ASD with those in patients with other psychiatric disorders, a previous meta-analysis of structural MRI studies showed that different cerebellar regions are affected in ASD and attention deficit hyperactivity disorder (Stoodley, 2014). Third, we did not use statistical methods to determine the sample size, but estimated it based on previous studies. The sample size of our original dataset was relatively small, and thus, the statistical power may not be high. While it is straightforward to calculate the sample size for a single outcome variable in typical biological studies, calculating the sample size for neuroimaging studies, such as an fMRI study, is a complicated process (Joyce and Hayasaka, 2012). Therefore, a few software tools for sample size calculation and power analysis in such studies were developed; these include Fmripower (Mumford and Nichols, 2008), PowerMap (Joyce and Hayasaka, 2012), and Neuropower (Durnez et al., 2016). However, these software tools are available only for task-fMRI studies, not rsFC MRI studies. To the best of our knowledge, no software tools for sample size calculation and power analysis in rsFC MRI studies have been developed. Thus, how the sample size in rsFC MRI studies must be calculated is an open question that has not yet been addressed. Although the sample size of our original dataset was relatively small, we partially replicated our findings in an independent larger dataset, which was larger than that used in previous cerebellar rsFC studies conducted in patients with ASD (Khan et al., 2015; Olivito et al., 2017).

## Conclusion

In conclusion, the present study reveals significantly increased and decreased FCs between the cerebellum and the cerebral cortices in children and adolescents with ASD. Findings of aberrant FCs converged primarily on the posterior hemisphere, the right dentate nucleus, and the posterior vermis of the cerebellum. In addition, our findings were partially replicated in an independent larger dataset. Our findings suggest that aberrant cerebellar–cerebral FCs are associated with motor, socio-communicative, and executive function issues in patients with ASD. These findings improve current knowledge regarding the neural substrates underlying the symptoms of ASD, and may help to provide effective therapeutic interventions in children and adolescents with ASD.

## Author Contributions

MKT and RH designed the study. IM, KK-S, and JM contributed to consensus clinical diagnosis. RH, JM, IH, FN, MYT, and YW conducted data collection. RH and JM performed the statistical analyses. RH, MKT, and TK drafted the manuscript. All authors contributed to writing the manuscript, and have read and approved the final manuscript.

## Conflict of Interest Statement

The authors declare that the research was conducted in the absence of any commercial or financial relationships that could be construed as a potential conflict of interest.

## Footnotes

1 http://fcon_1000.projects.nitrc.org/indi/abide/abide_II.html

2 http://www.nitrc.org/projects/conn

3 http://www.fil.ion.ucl.ac.uk/spm/software/spm12/

4 http://www.nitrc.org/projects/artifact_detect

5 http://www.mccauslandcenter.sc.edu/mricro/mricron/

## Abbreviations

ABIDE: Autism Brain Imaging Data Exchange
ADOS-2: Autism Diagnostic Observation Schedule Second Edition
ADOS-G: Autism Diagnostic Observation Schedule – Generic
AG: angular gyrus
ASD: autism spectrum disorder
BOLD: blood-oxygen-level-dependent
BRI: behavioral regulation index
BRIEF: Behavior Rating Inventory of Executive Function
CompCor: component-based noise correction
FC: functional connectivity
FD: framewise displacement
FDR: false discovery rate
FSIQ: full-scale intelligence quotient
GEC: global executive composite
GM: gray matter
GU: Georgetown University
IFG: inferior frontal gyrus
IPL: inferior parietal lobule
ITG: inferior temporal gyrus
KKI: Kennedy Krieger Institute
M-ABC: Movement Assessment Battery for Children
MCP: middle cerebellar peduncle
MdFG: medial frontal gyrus
MFG: middle frontal gyrus
MI: metacognition index
MNI: Montreal Neurological Institute
MRI: magnetic resonance imaging
MTG: middle temporal gyrus
NYU: New York University
ROIs: regions of interest
rsFC: resting state functional connectivity
rTMS: repetitive transcranial magnetic stimulation
SCI: social communication and interaction index
SCP: superior cerebellar peduncle
SDSU: San Diego State University
SMG: supramarginal gyrus
SPL: superior parietal lobule
SRS: Social Responsiveness Scale
TD: typically developing
