# Supplementary material for: Aberrant Cerebellar–Cerebral Functional Connectivity in Children and Adolescents With Autism Spectrum Disorder
Source: Front Hum Neurosci. 2018 Nov 13;12:454. doi: 10.3389/fnhum.2018.00454 (PMC6243023; doi:10.3389/fnhum.2018.00454)
Supplement: Supplementary file 1 [file Data_Sheet_1.docx]

***Supplementary Material***

**Aberrant Cerebellar-Cerebral Functional Connectivity in Children and Adolescents with Autism Spectrum Disorder**

**Ryuzo Hanaie, Ikuko Mohri, Kuriko Kagitani-Shimono, Masaya Tachibana, Junko Matsuzaki, Ikuko Hirata, Fumiyo Nagatani, Yoshiyuki Watanabe, Taiichi Katayama, Masako Taniike^*^**

***Correspondence:**

Masako Taniike

[masako@kokoro.med.osaka-u.ac.jp](mailto:masako@kokoro.med.osaka-u.ac.jp)

1. **Supplementary Figures and Tables**
   1. **Supplementary Tables**

**Table S1.** Demographic characteristics of the participants in the replication dataset

ASD: autism spectrum disorder, TD: typically developing controls, FSIQ = full-scale intelligence quotient, ADOS-G: Autism Diagnostic Observation Schedule—Generic, ADOS-2: Autism Diagnostic Observation Schedule Second Edition, FD: framewise displacement, SD: standard deviation

^a^ADOS-G total scores were available for 2 participants with ASD in the GI dataset.

^b^ADOS-2 total scores were available for 2 participants with ASD in the GI dataset.

^c^ADOS-G total scores were available for 6 participants with ASD in the KKI dataset.

^d^ADOS-2 total scores were available for 11 participants with ASD in the KKI dataset.

^e^Comorbidity included simple phobia and attention deficit hyperactivity, generalized anxiety, oppositional defiant, and obsessive compulsive disorders in the KKI dataset.

^f^ADOS-G total scores were available for 9 participants with ASD in the NYU_1 dataset, and these participants were also administered ADOS 2.

^g^ADOS-2 total scores were available for 11 participants with ASD in the NYU_1 dataset.

^h^Comorbidity included attention deficit hyperactivity, generalized anxiety, oppositional defiant, tic, and Tourette’s disorders in the NYU_1 dataset.

^i^ADOS-2 total scores were available for 10 participants with ASD in the SDSU dataset.

^j^ADOS-G total scores were available for 17 participants with ASD in the total sample, and 9 participants with ASD were also administered ADOS 2.

^k^ADOS-2 total scores were available for 34 participants with ASD in the total sample population.

**p* < 0.05, ***p* < 0.01

**Table S2.** Behavioral measurement scores for the replication dataset

ASD: autism spectrum disorder, TD: typically developing participants, SD: standard deviation

^a^Data from the Social Responsiveness Scale were available for 31 participants with ASD and 59 TD participants.

^b^Data from the Social Responsiveness Scale 2 were available for 11 participants with ASD and 29 TD participants.

## Supplementary Figures


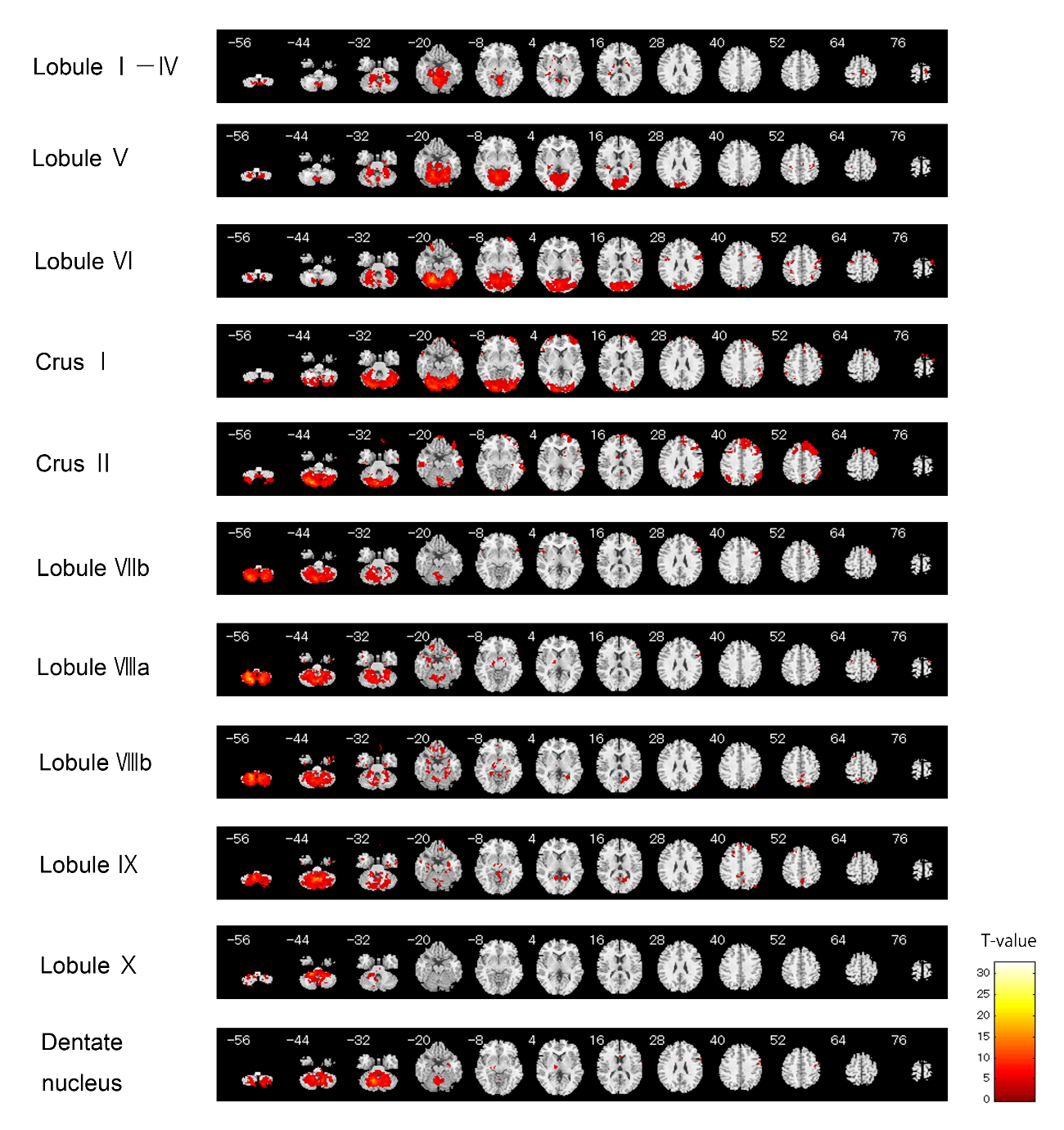


**Figure S1.** Brain regions showing positive functional connectivity with the left cerebellar hemisphere and the dentate nucleus of the cerebellum in the typically developing group. All results were thresholded using a voxel-height threshold at uncorrected *p* < 0.001 with cluster-extent threshold at false discovery rate-corrected *p* < 0.05. Images of results were created using the xjview toolbox (<http://www.alivelearn.net/xjview>).


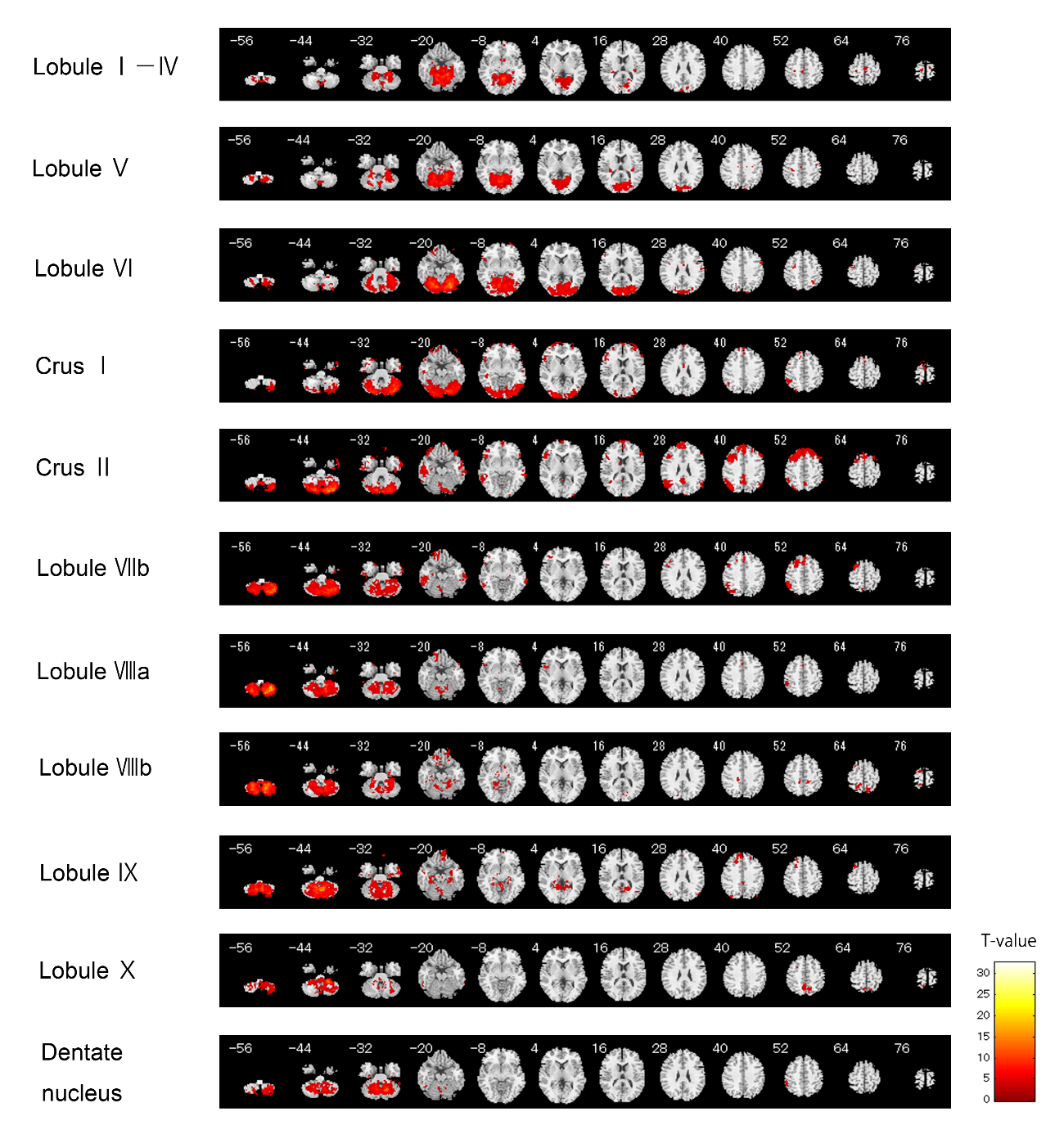


**Figure S2.** Brain regions showing positive functional connectivity with the right cerebellar hemisphere and the dentate nucleus of the cerebellum in the typically developing group. All results were thresholded using a voxel-height threshold at uncorrected *p* < 0.001 with cluster-extent threshold at false discovery rate-corrected *p* < 0.05. Images of results were created using the xjview toolbox (<http://www.alivelearn.net/xjview>).


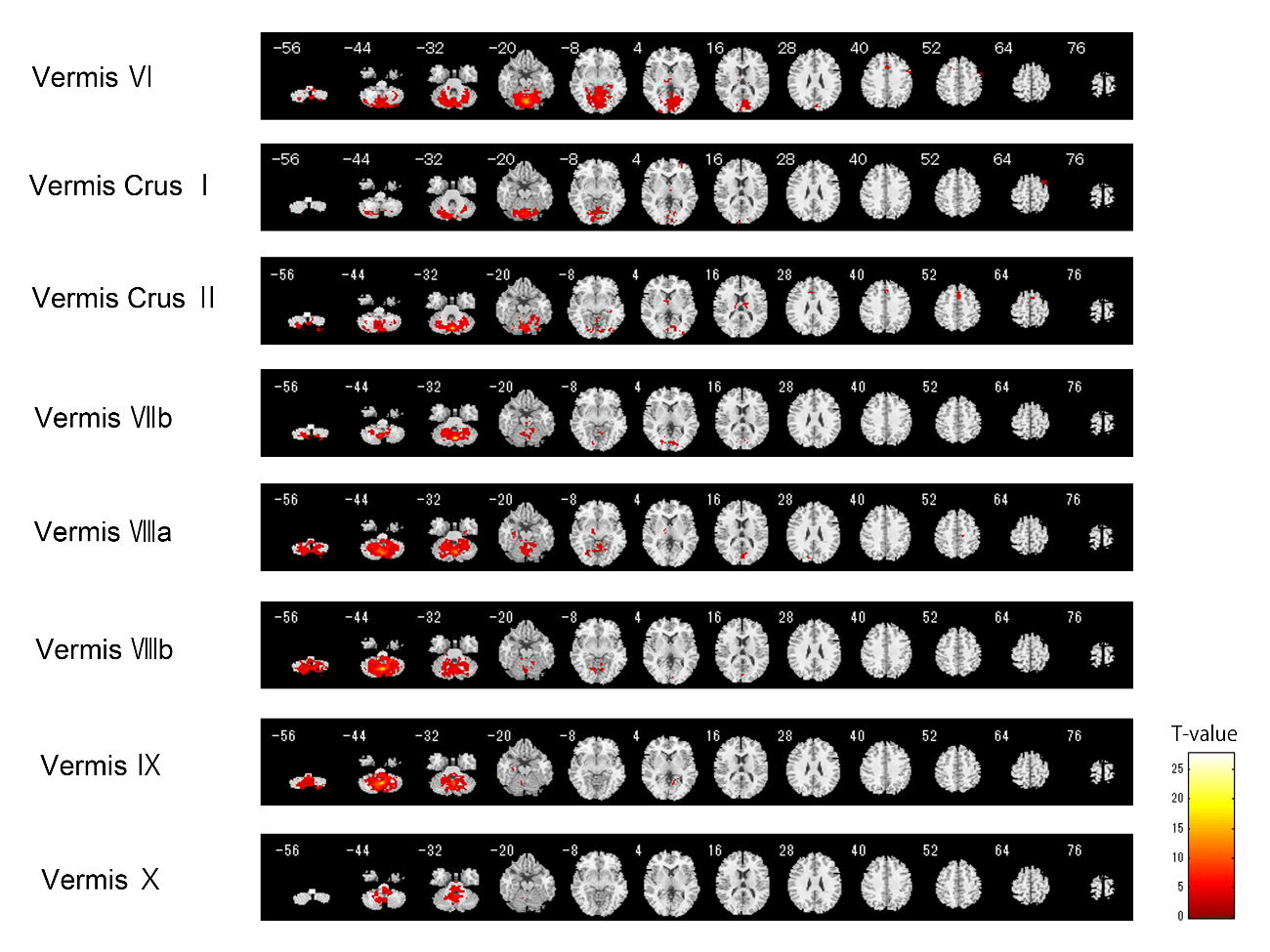


**Figure S3.** Brain regions showing positive functional connectivity with the cerebellar vermis in the typically developing group. All results were thresholded using a voxel-height threshold at uncorrected *p* < 0.001 with cluster-extent threshold at false discovery rate-corrected *p* < 0.05. Images of results were created using the xjview toolbox (<http://www.alivelearn.net/xjview>).


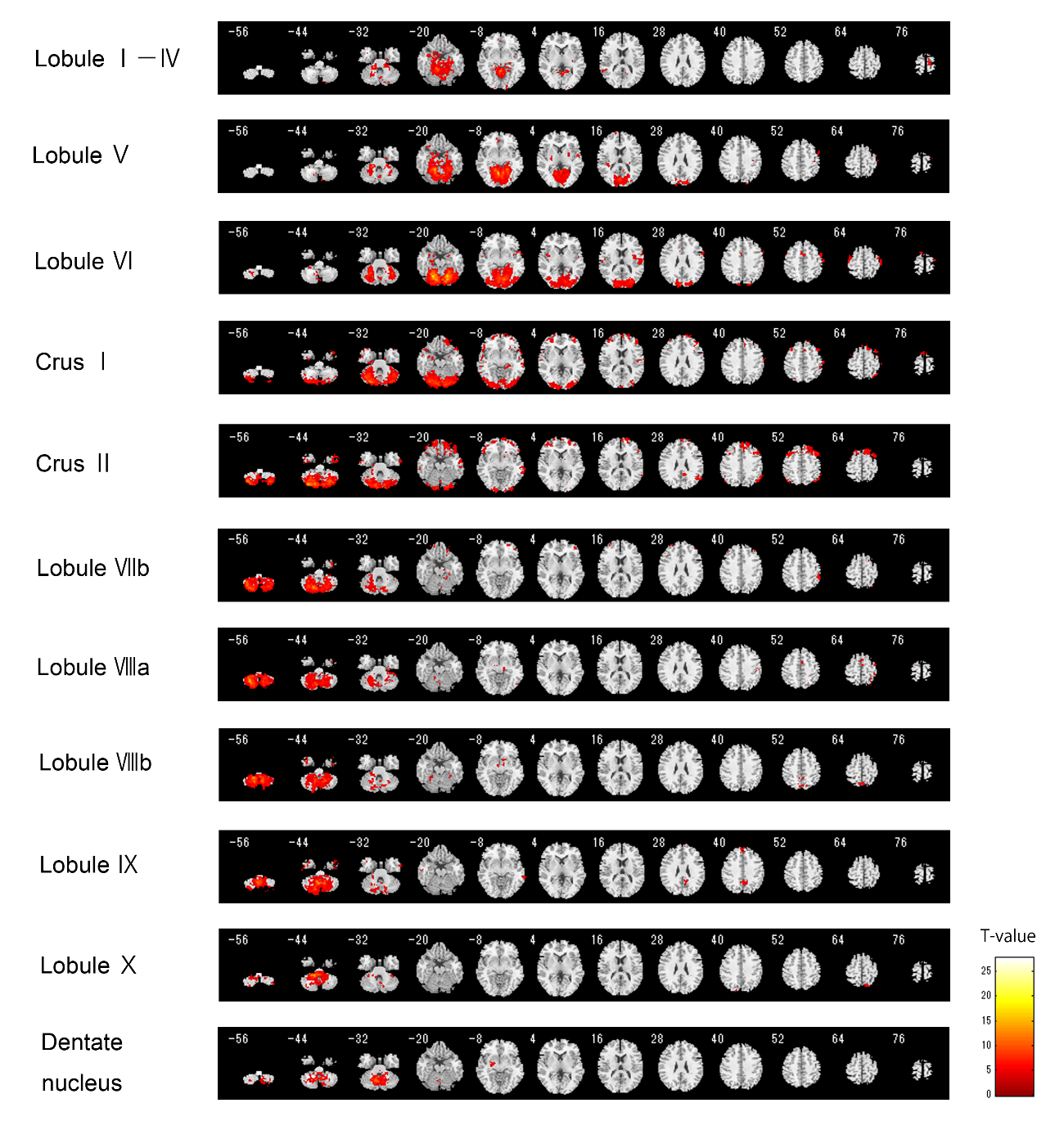


**Figure S4.** Brain regions showing positive functional connectivity with the left cerebellar hemisphere and the dentate nucleus of the cerebellum in the autism spectrum disorder group. All results were thresholded using a voxel-height threshold at uncorrected p < 0.001 with cluster-extent threshold at false discovery rate-corrected p < 0.05. Images of results were created using the xjview toolbox (<http://www.alivelearn.net/xjview>).


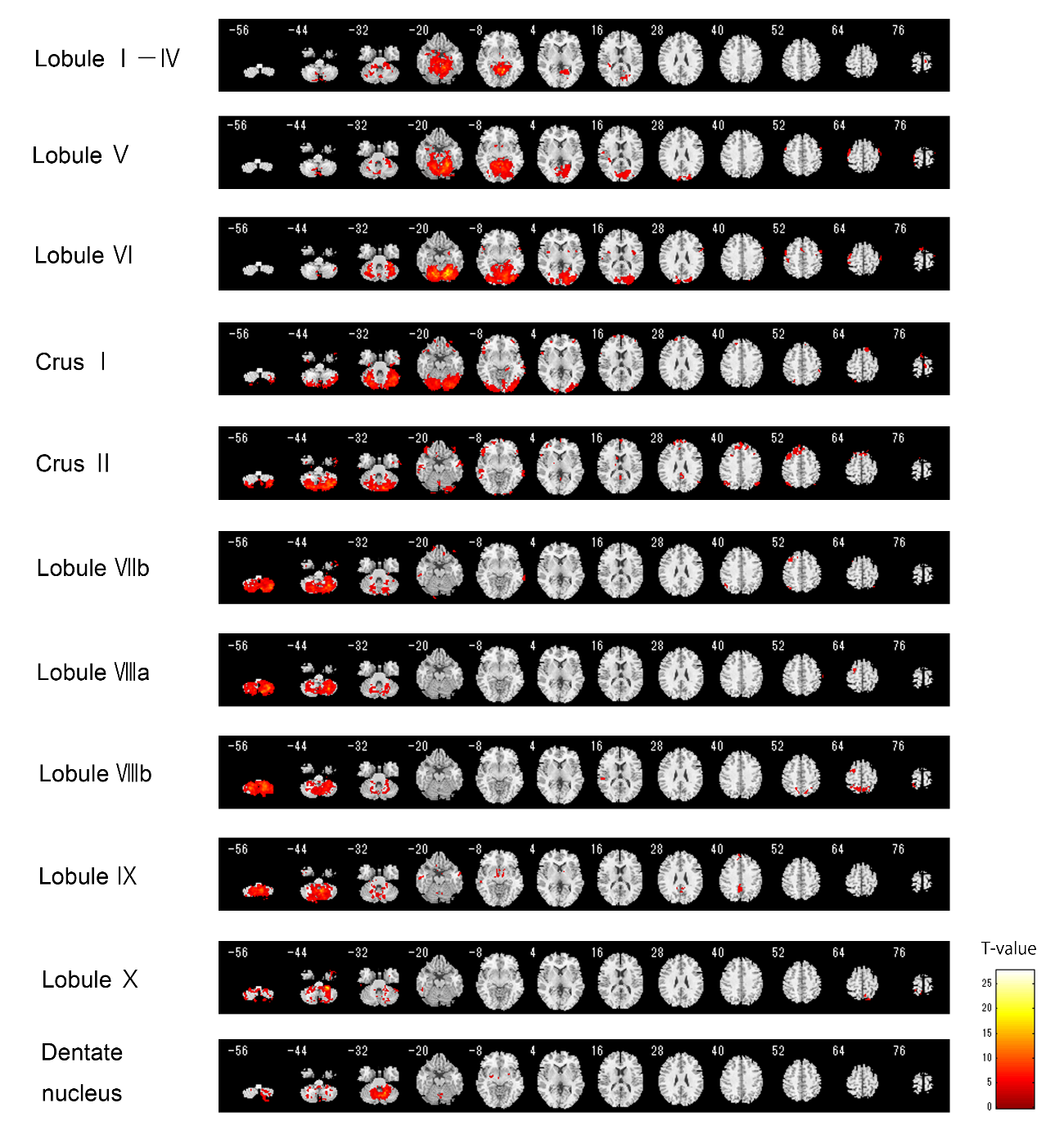


**Figure S5.** Brain regions showing positive functional connectivity with the right cerebellar hemisphere and the dentate nucleus of the cerebellum in the autism spectrum disorder group. All results were thresholded using a voxel-height threshold at uncorrected *p* < 0.001 with cluster-extent threshold at false discovery rate-corrected *p* < 0.05. Images of results were created using the xjview toolbox (<http://www.alivelearn.net/xjview>).


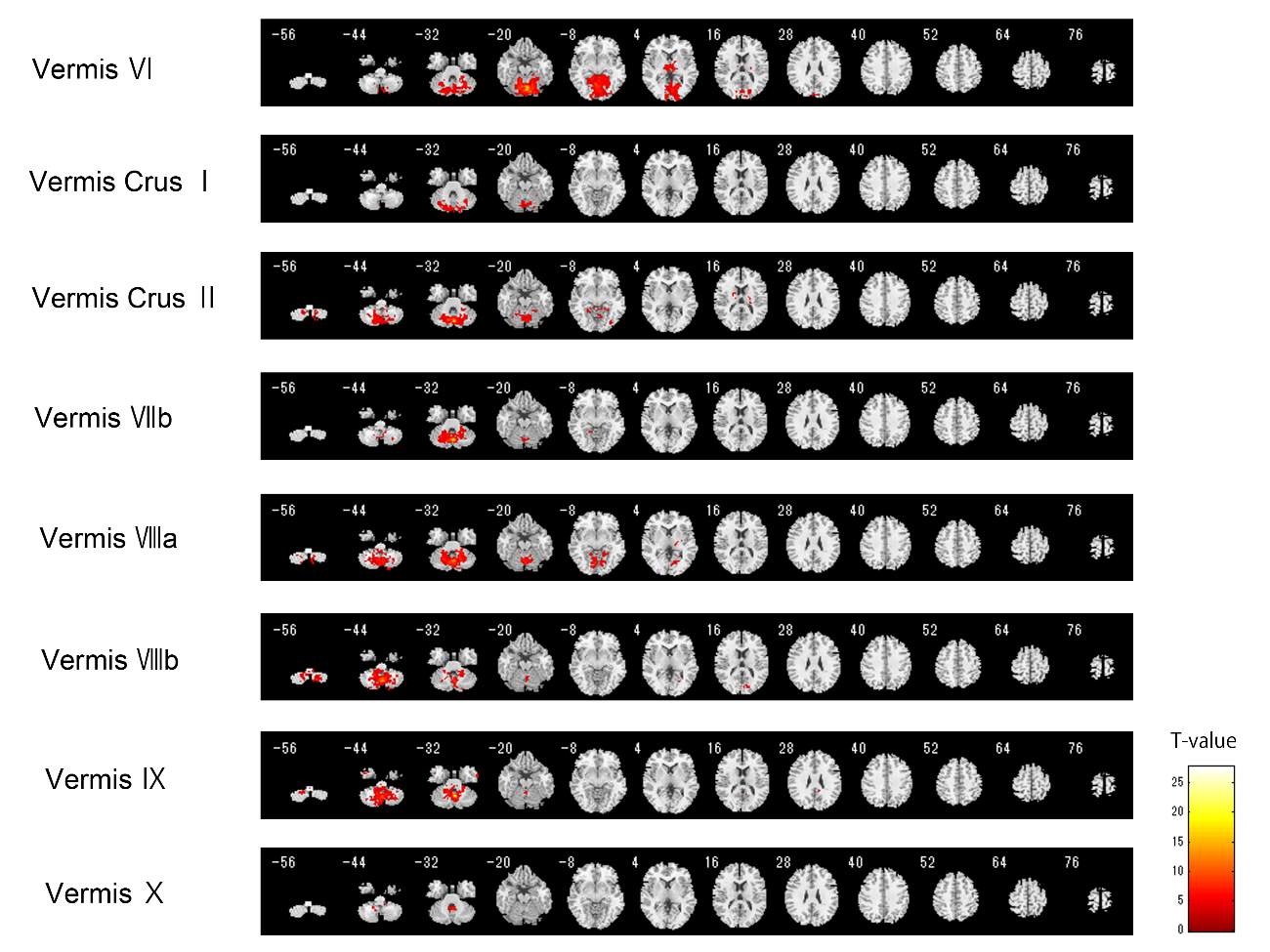


**Figure S6.** Brain regions showing positive functional connectivity with the cerebellar vermis in the autism spectrum disorder group. All results were thresholded using a voxel-height threshold at uncorrected *p* < 0.001 with cluster-extent threshold at false discovery rate-corrected *p* < 0.05. Images of results were created using the xjview toolbox (<http://www.alivelearn.net/xjview>).


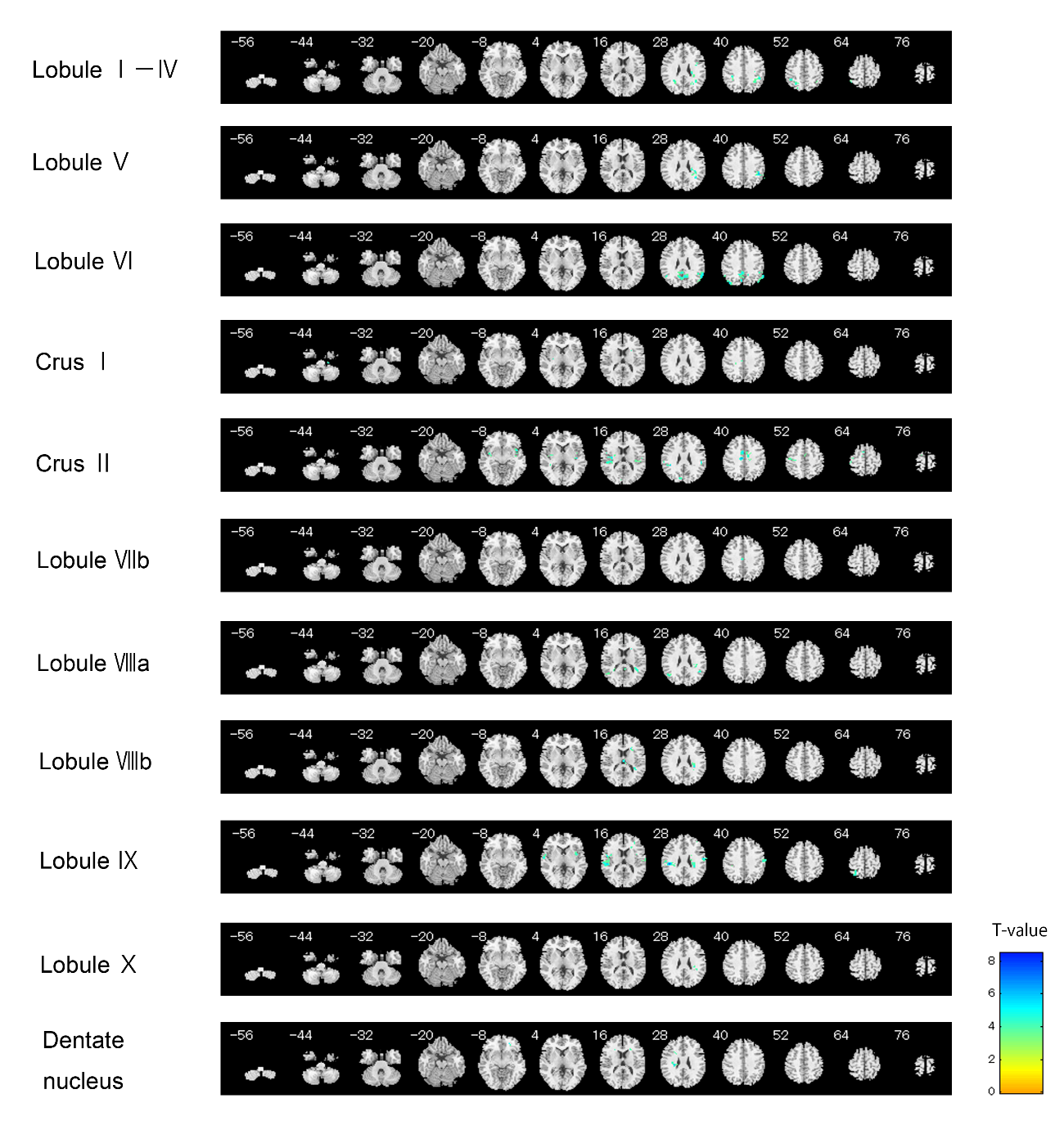


**Figure S7.** Brain regions showing negative functional connectivity with the left cerebellar hemisphere and the dentate nucleus of the cerebellum in the typically developing group. All results were thresholded using a voxel-height threshold at uncorrected *p* < 0.001 with cluster-extent threshold at false discovery rate-corrected *p* < 0.05. Images of results were created using the xjview toolbox (<http://www.alivelearn.net/xjview>).


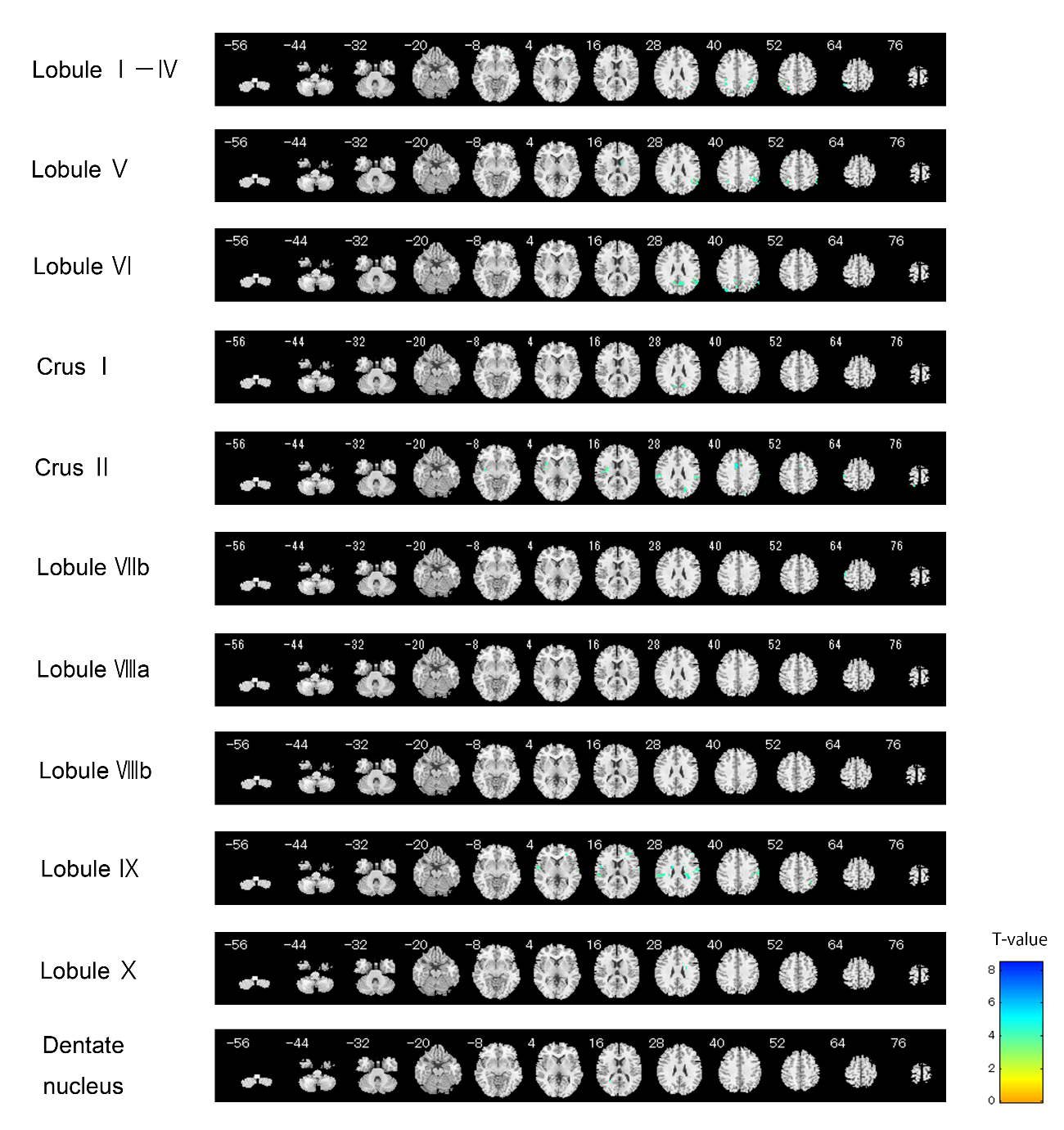


**Figure S8.** Brain regions showing negative functional connectivity with the right cerebellar hemisphere and the dentate nucleus of the cerebellum in the typically developing group. All results were thresholded using a voxel-height threshold at uncorrected *p* < 0.001 with cluster-extent threshold at false discovery rate-corrected *p* < 0.05. Images of results were created using the xjview toolbox (<http://www.alivelearn.net/xjview>).


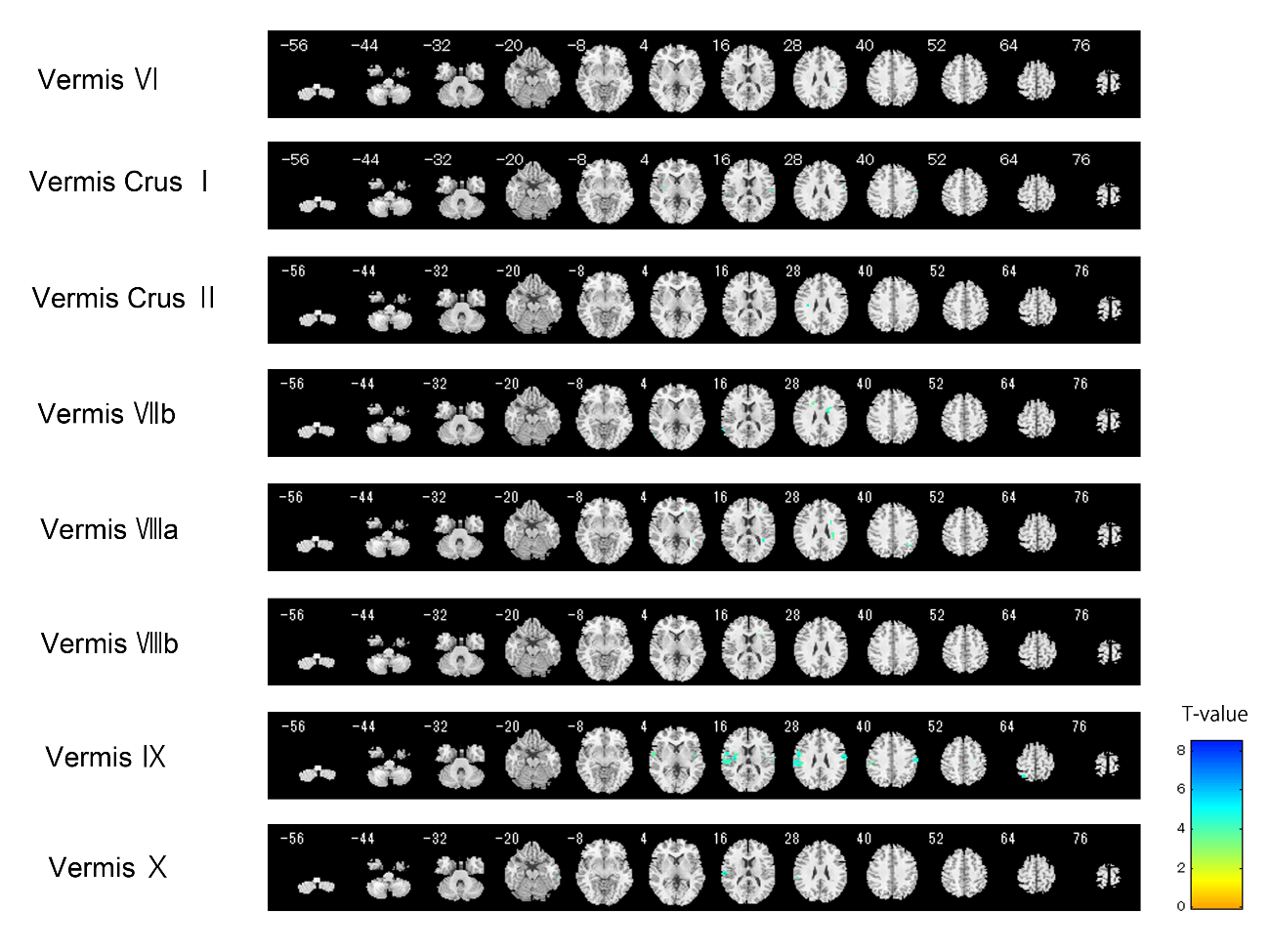


**Figure S9.** Brain regions showing negative functional connectivity with the cerebellar vermis in the typically developing group. All results were thresholded using a voxel-height threshold at uncorrected *p* < 0.001 with cluster-extent threshold at false discovery rate-corrected *p* < 0.05. Images of results were created using the xjview toolbox (<http://www.alivelearn.net/xjview>).


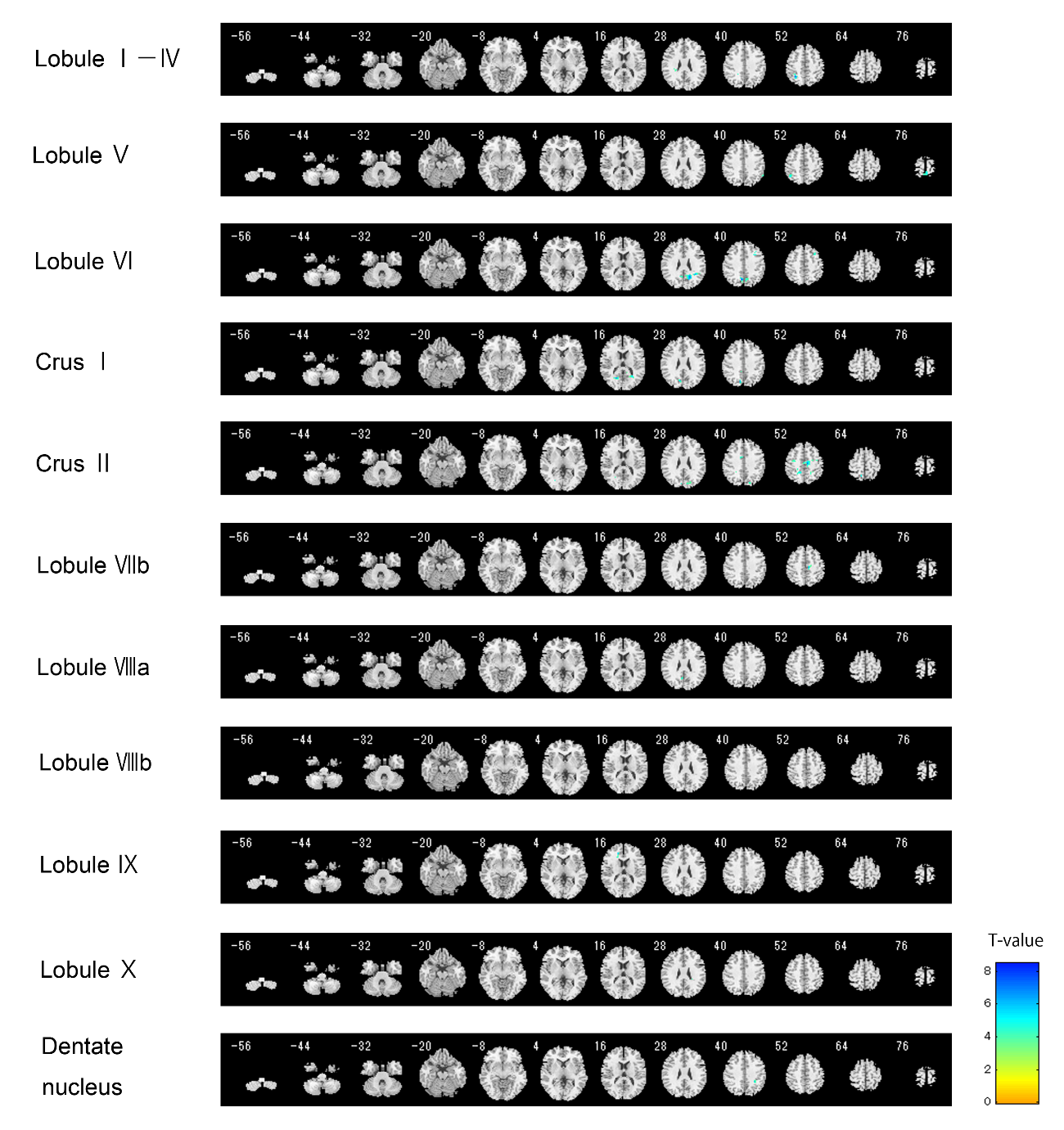


**Figure S10.** Brain regions showing negative functional connectivity with the left cerebellar hemisphere and the dentate nucleus of the cerebellum in the autism spectrum disorder group. All results were thresholded using a voxel-height threshold at uncorrected *p* < 0.001 with cluster-extent threshold at false discovery rate-corrected *p* < 0.05. Images of results were created using the xjview toolbox (<http://www.alivelearn.net/xjview>).


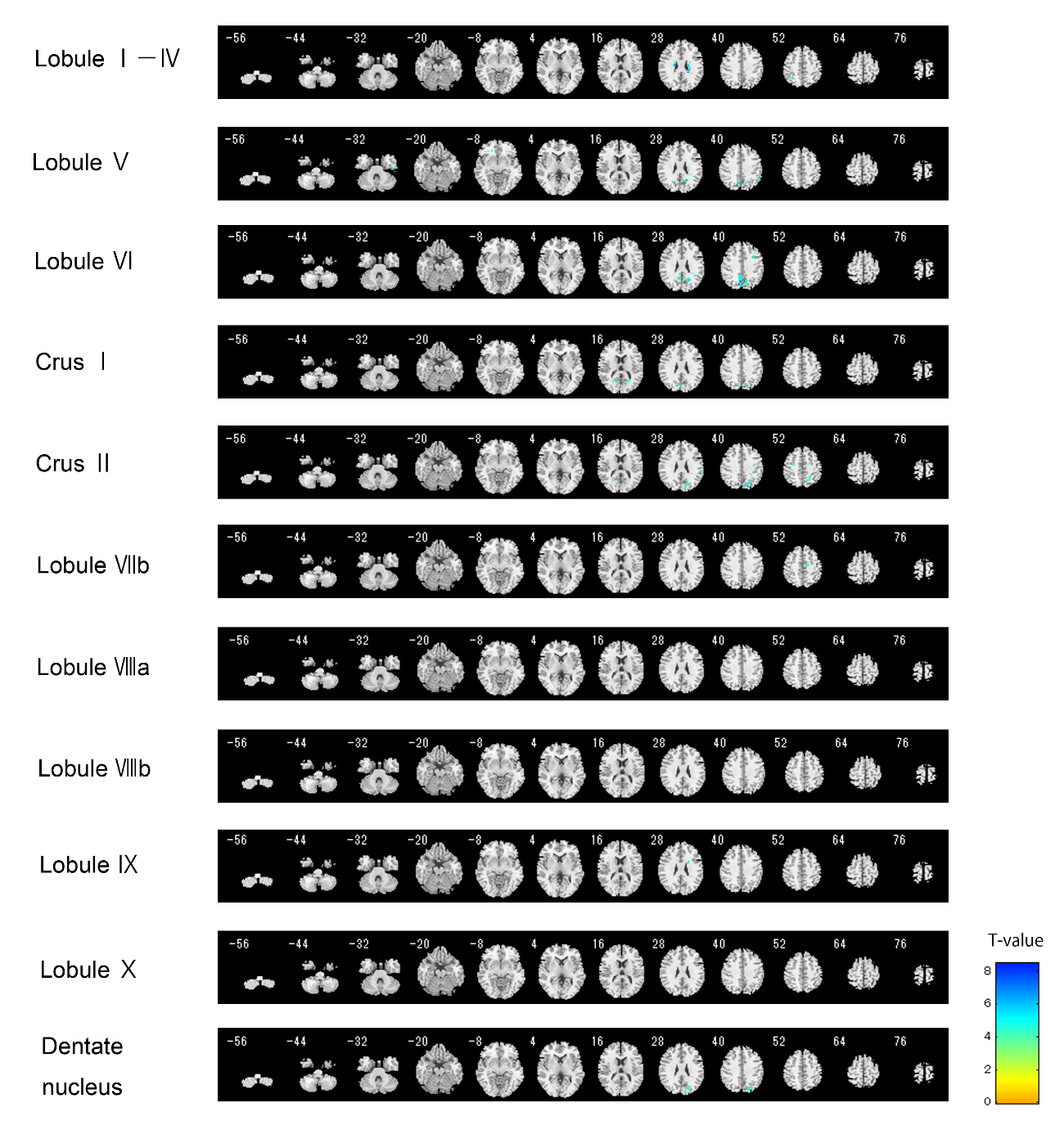


**Figure S11.** Brain regions showing negative functional connectivity with the right cerebellar hemisphere and the dentate nucleus of the cerebellum in the autism spectrum disorder group. All results were thresholded using a voxel-height threshold at uncorrected *p* < 0.001 with cluster-extent threshold at false discovery rate-corrected *p* < 0.05. Images of results were created using the xjview toolbox (<http://www.alivelearn.net/xjview>).


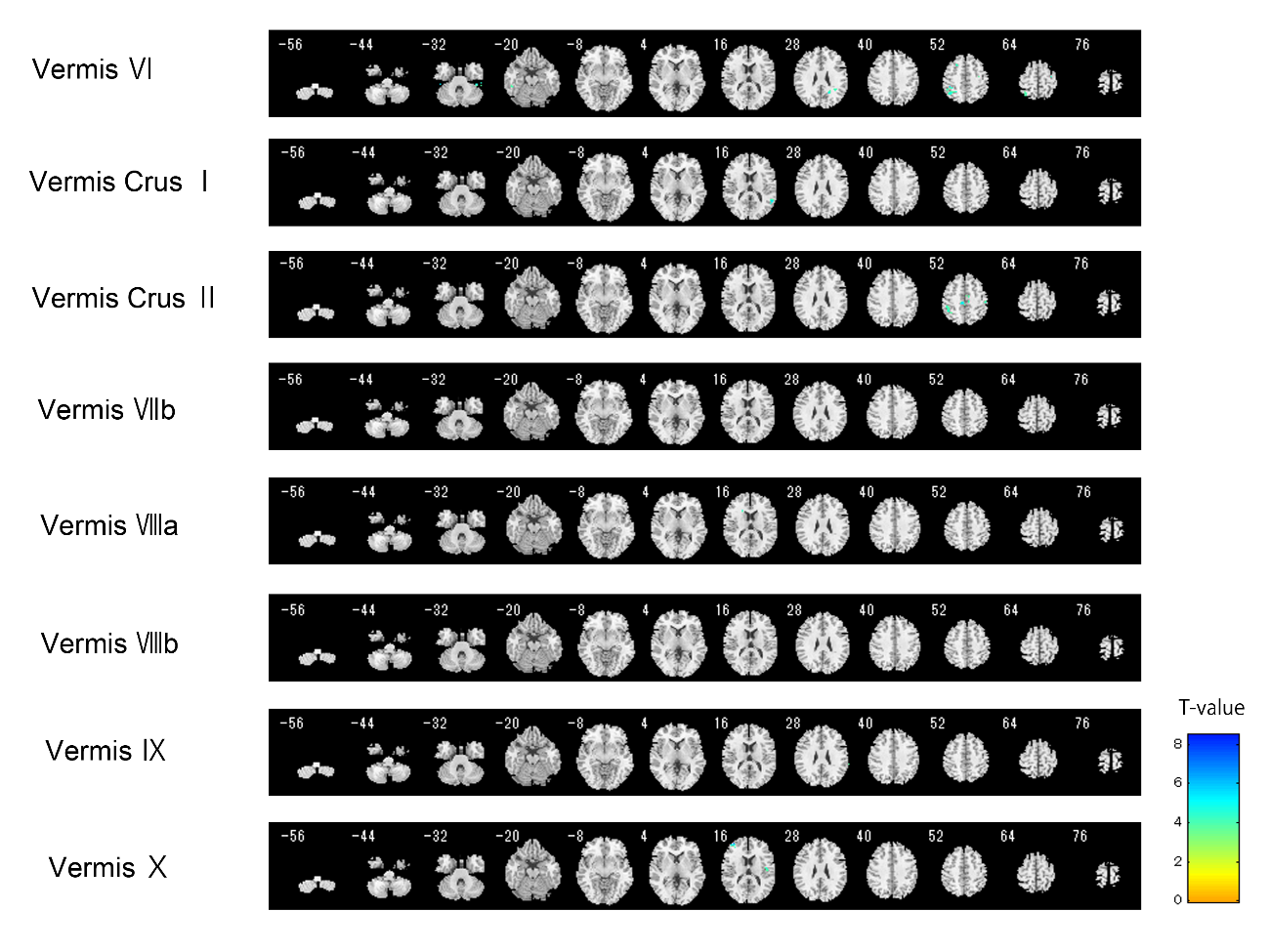


**Figure S12.** Brain regions showing negative functional connectivity with the cerebellar vermis in the autism spectrum disorder group. All results were thresholded using a voxel-height threshold at uncorrected *p* < 0.001 with cluster-extent threshold at false discovery rate-corrected *p* < 0.05. Images of results were created using the xjview toolbox (<http://www.alivelearn.net/xjview>).
